# Association of *MTHFR 677C>T* Polymorphism with Susceptibility to Ovarian and Cervical Cancers: A Systematic Review and Meta-Analysis

**DOI:** 10.31557/APJCP.2019.20.9.2569

**Published:** 2019

**Authors:** Mojgan Karimi-Zarchi, Mansour Moghimi, Hajar Abbasi, Amaneh Hadadan, Erfaneh Salimi, Majid Morovati-Sharifabad, Mohammad Javad Akbarian-Bafghi, Masoud Zare-Shehneh, Alireza Mosavi-Jarrahi, Hossein Neamatzadeh

**Affiliations:** 1 *Department of Gynecology and Obstetrics, Iran University of Medical Sciences,*; 3 *Department of Gynecology and Obstetrics,*; 7 *Department of Social Medicine, Medical School, Shahid Beheshti University of Medical Sciences, Tehran, *; 2 *Department of Pathology,*; 6 *Department of Medical Genetics, *; 8 *Mother and Newborn Health Research Center, Shahid Sadoughi University of Medical Sciences, Yazd,*; 4 *Department of Basic Science, Faculty of Veterinary Medicine, Ardakan University, Ardakan,*; 5 *Department of Healthcare Management, Bam University of Medical Sciences, Bam, Iran. *

**Keywords:** Ovarian cancer, cervical cancer, *MTHFR* gene, polymorphism, meta-analysis

## Abstract

**Background:**

Previous studies have evaluated the impact of *MTHFR 677C>T* polymorphism on susceptibility to ovarian and cervical cancers in women, but the conclusions are still controversial. To get a more precise evaluation of the association between *MTHFR 677C>T* polymorphism and risk of ovarian and cervical cancers, we performed a meta-analysis of the association of all eligible studies.

**Methods:**

A comprehensive search performed in PubMed, Google Scholar, CNKI, and Web of Science databases to identify the relevant studies up to October 15, 2018. The strength of the association was estimated by odds ratios (OR) with 95% confidence interval (CI).

**Results:**

A total of 27 case-control studies including eleven studies with 4990 cases 7730 controls on ovarian cancer and 16 studies with 4990 cases and 7730 controls on cervical cancer were selected. Pooled data revealed that the *MTHFR 677C>T* polymorphism not significantly associated with an increased risk of ovarian and cervical cancers under all five genetic models. However, stratified analysis by ethnicity showed that the *MTHFR 677C>T* polymorphism was significantly associated with risk of ovarian cancer in Asians. No publication bias was found in the current meta-analysis.

**Conclusions:**

The results of this meta-analysis proposes that the *MTHFR 677C>T* polymorphism may not play a role in development of ovarian and cervical cancers in overall population. Further well-designed studies are necessary to clarify the precise role of the *MTHFR 677C>T* polymorphism on ovarian and cervical cancers risk.

## Introduction

Gynecological cancers are among the most common cancers in women and hence a major health problem worldwide (Maheshwari et al., 2016). Ovarian and cervical cancers are the most common gynecological cancers affecting women worldwide (Torre et al., 2017). Ovarian cancer is the second most common cancer and main cause of death with gynecological tumors worldwide (Qin et al., 2013; Zhu and Sun, 2017). Moreover, cervical cancer is the third most frequent neoplasm among women worldwide (Rocha et al., 2017). In 2017, a study showed that ovarian cancer (47%) followed by cervical cancer (29%) are the most common gynecological malignancy among Pakistan women (Manzoor et al., 2017). Despite continuous advances in cancer biology research, the etiology of ovarian and cervical cancer are not known exactly, partly because of the inconsistency of findings among epidemiological studies (Yu et al., 2013). A reappraisal of Genome-wide association studies (GWAS) and genetic association studies suggested a strong genetic component to susceptibility to ovarian and cervical cancers (Fearon et al., 2013).

Epidemiological studies had identified that Methylene tetrahydrofolate reductase (MTHFR) was a potential genetic marker of different malignancies (Yi et al., 2016; He and Shen, 2017). The human MTHFR gene is located on chromosome 1p36.3, consist of 11 exons and spans 2.2 kb of genomic DNA (Abedinzadeh et al., 2015; Azarpira et al., 2018). It is encodes the vital enzyme which plays a key role in the folate/homocysteine metabolic pathway and regulates the intracellular folate level for the synthesis and methylation of DNA (Azarpira et al., 2018; Kamali et al., 2018). In humans, the *MTHFR 677C>T* (in exon 4) polymorphism has been heavily studied in different disease. The *MTHFR 677C>T* is associated with reduced enzyme activity and arise an elevated plasma homocysteine level. Moreover, the *MTHFR 677C>T* polymorphism leads to increased heat liability and reduced enzymatic capability for methylation of Homocysteine (Rozycka et al., 2014). The mutant homozygous genotype (TT) of *MTHFR 677C>T* polymorphism was particularly common in northern China (20%), southern Italy (26%), and Mexico (32%) (Wilcken, 2003).

Over the past decades, a large number of epidemiological studies and meta-analyses have evaluated the association between the *MTHFR 677C>T* polymorphism and susceptibility to ovarian and cervical cancer (Yi et al., 2016; He and Shen, 2017). However, the results were conflicting and inconclusive, presumably due to small sample size in each published study, various genetic backgrounds and possible selection bias (He and Shen, 2017). Subsequently, a few novel studies have recently been performed to estimate the associations of *MTHFR 677C>T* polymorphism with risk of ovarian and cervical cancer and provide new evidences that were not included in the previous meta-analyses. Thus, this meta-analysis covering all potentially eligible studies was performed to get a more precise evaluation of the association between *MTHFR 677C>T* polymorphism and risk of ovarian and cervical cancers.

## Materials and Methods

*Literature Search Strategy*

A comprehensive searched in PubMed, Google scholar, Web of Science, EMBASE, Chinese Biomedical database, and China National Knowledge Infrastructure (CNKI) databases was performed to obtain the all relevant studies investigated association of *MTHFR 677C>T* polymorphism with risk of ovarian and cervical cancers up to October 15, 2018. The following keywords and terms were used: (‘’Gynecological Cancer’’ OR ‘’Ovarian Cancer’’ OR ‘’Epithelial Ovarian Cancer’’ OR ‘’Cervical Cancer’’) AND (‘’Methylene tetrahydrofolate reductase’’ OR ‘’MTHFR’’ OR ‘’677C>T’’ OR ‘’rs1801133’’) AND (“Polymorphism” OR ‘’SNP’’ OR ‘’Mutation’’ OR “Variant” OR “Variation”). Moreover, the references of the retrieved articles manually checked for other potential studies that possibly have been missed in the initial search.

*Inclusion Criteria and Data Extraction*

Studies were included in the current meta-analysis only if they met all of the following criteria: a) studies with case-control or cohort design; b) only published studies; c) evaluated the association of *MTHFR 677C>T* polymorphism with ovarian cancer and cervical cancer; and d) the number of *MTHFR 677C>T* polymorphism genotypes in the cases and healthy control was reported to estimates odds ratio (OR) and 95% confidence interval (CI). The exclusion criteria were: a) abstracts, case reports, reviews, previous meta-analyses, posters, letters to editor and commentaries; b) animal studies; c) case only studies; d) linkage or sibling studies; e) studies did not calculated *MTHFR* polymorphisms genotype frequencies or which the number of genotypes and alleles could not be ascertained; f) studies on other polymorphisms of *MTHFR* gene; and g) overlapping studies and studies duplicate or containing previously published data. Moreover, if studies had overlapping data, only the study with the largest population or more recently published data was finally selected.

*Data Extraction*

Two authors (H.A and A.H) independently assessed the articles for their eligibility for inclusion and the needed data were carefully extracted based on the inclusion criteria above using a standard form. Any disagreements were solved by discussion with a third author (E.S). The following data were extracted for each study: first author’s name, publication year, country, ethnicity, source of control (hospital-based or population-based), genotyping methods, and the number of alleles and genotypes in the cases and controls, minor allele frequency (MAF) among controls, and P-value for Hardy-Weinberg equilibrium (HWE).

**Figure 1:**
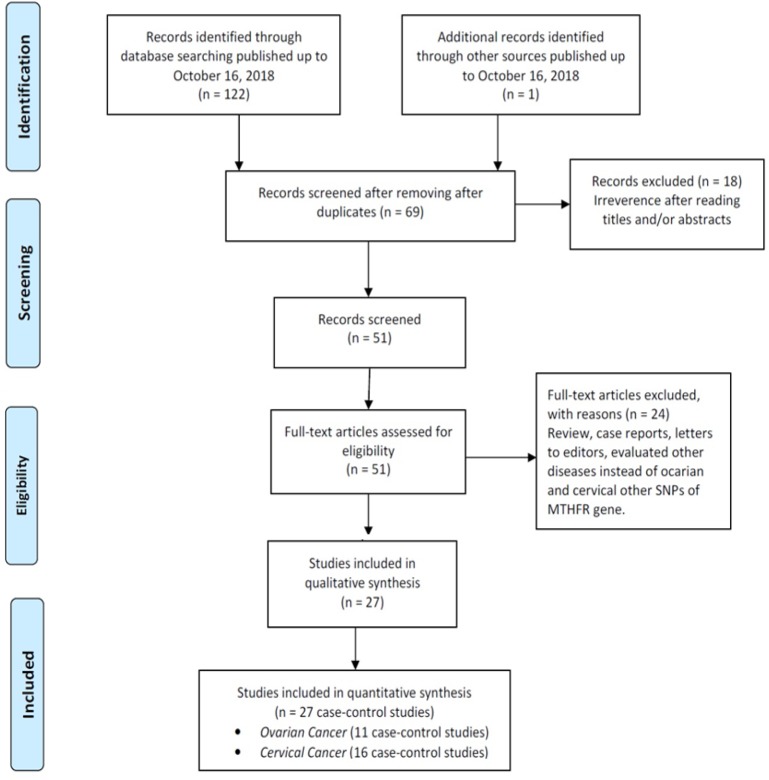
The Flow Diagram of the Included and Excluded Studies

**Figure 2:**
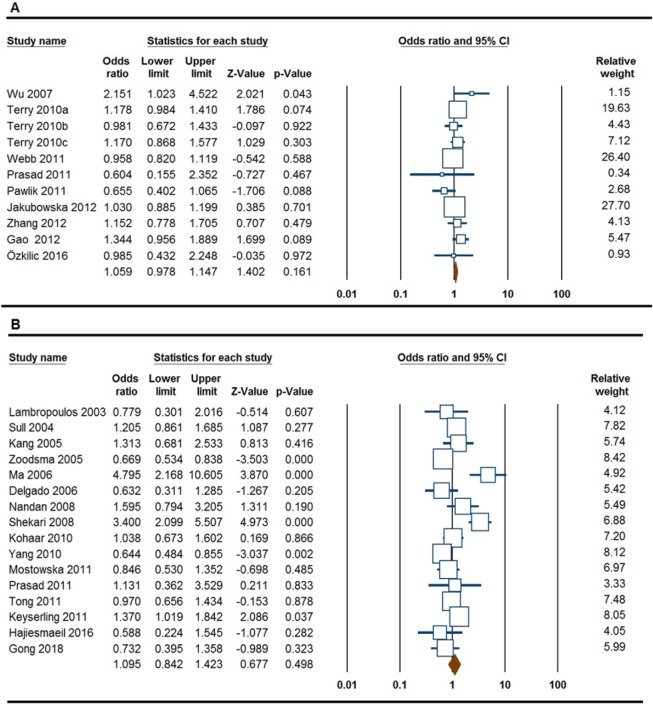
Forest Plot for the Association of *MTHFR 677C>T* Polymorphism with Risk of Ovarian and Cervical Cancer. A, ovarian cancer (heterozygote model, TC vs. CC); B, cervical cancer (dominant model, TT+TC vs. CC)

**Figure 3:**
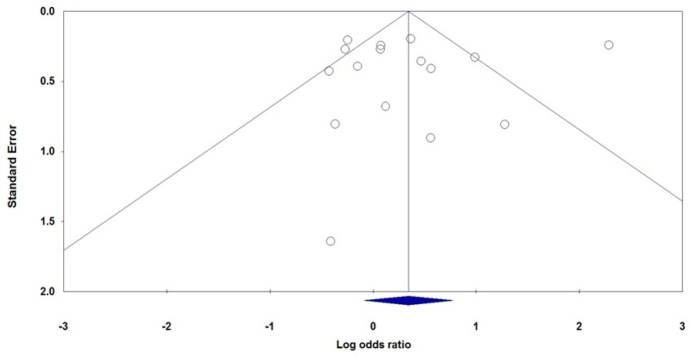
Begg’s Funnel Plot for Association between *MTHFR 677C>T* Polymorphism and Cervical Cancer Risk under the Recessive Genetic Model (TT vs. TC+CC).

**Table 1:** Characteristics of Studies Included in the Meta-analysis

First Author	Country (Ethnicity)	SOC	Genotyping Technique	Case/Control	Cases					Controls					MAFs	HWE
					Genotypes			Allele		Genotypes			Allele			
Ovarian Cancer					CC	CT	TT	C	T	CC	CT	TT	C	T		
Wu 2007	China (Asian)	HB	NA	81/8	17	40	24	74	88	32	35	13	99	61	0.381	0.515
Terry 2010	USA (Caucasian)	HB	TaqMan	1059/1125	427	492	140	1346	772	499	488	138	1486	764	0.340	0.270
Terry 2010	USA (Caucasian)	HB	TaqMan	158/496	71	72	10	184	92	210	217	55	637	327	0.339	0.925
Terry 2010	USA (Caucasian)	HB	TaqMan	364/412	164	167	33	495	233	193	168	51	554	270	0.328	0.130
Webb 2011	Australia (Caucasian)	PB	MassARRAY	1638/1278	744	709	185	2197	1079	571	568	139	1710	846	0.331	0.898
Prasad 2011	India (Asian)	HB	PCR-PFLP	80/125	72	3	5	147	13	116	8	1	240	10	0.040	0.062
Pawlik 2011	Poland (Caucasian)	PB	PCR-PFLP	136/16	67	55	13	189	81	63	79	18	205	115	0.359	0.360
Jakubowska 2012	Poland (Caucasian)	HB	PCR-PFLP	985/335	423	446	116	1292	678	1447	1481	422	4375	2325	0.347	0.156
Zhang 2012	China (Asian)	HB	PCR-PFLP	215/218	102	94	19	298	132	115	92	11	322	114	0.261	0.170
Gao 2012	China (Asian)	HB	PCR-PFLP	224/432	97	100	27	294	154	232	178	22	642	222	0.257	0.100
Özkılıç 2016	Turkey (Caucasian)	HB	PCR-PFLP	50/54	18	28	4	258	36	19	30	5	68	40	0.370	0.160
Total				4990/773	2202	2206	450	6610	3106	3497	3344	875	10338	5094	0.330	0.077
Cervical Cancer					CC	CT	TT	C	T	CC	CT	TT	C	T		
Lambropoulos 2003	Greece (Caucasian)	HB	PCR-PFLP	21/91	11	8	2	30	12	42	37	12	121	61	0.335	0.403
Sull 2004	Korea (Asian)	HB	SNapShot	246/454	73	115	58	261	231	153	221	80	527	381	0.420	0.989
Kang 2005	Korea (Asian)	HB	PCR-PFLP	79/74	27	32	20	86	72	30	32	12	92	56	0.378	0.487
Zoodsma 2005	Netherlands (Caucasian)	HB	TaqMan	636/592	357	230	49	944	328	273	262	57	808	376	0.318	0.608
Ma 2006	China (Asian)	HB	PCR-PFLP	111/111	20	53	38	93	129	33	60	18	126	96	0.432	0.286
Delgado 2006	Mexico (Mixed)	HB	PCR-PFLP	70/89	18	34	14	70	62	20	49	20	89	69	0.500	0.340
Nandan 2008	India (Asian)	HB	PCR-PFLP	62/77	36	0	26	72	52	53	0	24	106	48	0.312	≤0.001
Shekari 2008	India (Asian)	HB	PCR-PFLP	200/2	125	68	7	318	82	170	28	2	368	32	0.080	0.489
Kohaar 2010	India (Asian)	HB	SNapShot	164/231	113	47	4	273	55	161	65	5	387	75	0.162	0.598
Yang 2010	China (Asian)	HB	PCR-PFLP	391/382	229	85	77	530	234	182	166	34	536	234	0.306	0.658
Mostowska 2011	Poland (Caucasian)	HB	PCR-PFLP	124/168	56	59	9	171	77	69	81	18	219	117	0.348	0.420
Prasad 2011	India (Asian)	PB	PCR-PFLP	62/125	57	5	0	119	5	116	8	1	240	10	0.040	0.062
Tong 2011	Korea (Asian)	HB	TaqMan	146/427	53	65	28	171	121	152	198	77	502	342	0.412	0.373
Keyserling 2011	Germany (Caucasian)	HB	LDR-PCR	386/328	164	188	34	516	256	165	136	27	466	190	0.290	0.890
Hajiesmaeil 2016	Iran (Asian)	HB	PCR-PFLP	22/74	13	7	2	21	11	34	36	4	104	44	0.297	0.157
Gong 2018	China (Asian)	HB	TaqMan	146/11	34	70	42	138	154	20	52	38	92	128	0.582	0.764
Total				2866/3533	1386	1066	410	3813	1881	1673	1431	429	4783	2259	0.324	≤0.001

Abbreviation, SOC, Source Of Control; HB, Hospital Based; PB, Population Based; NA, Not applicable; PCR-RFLP, Polymerase Chain Reaction-Restriction Fragment Length Polymorphism; AS-PCR, Allele-Specific PCR; MAF, Minor Allele Frequency; HWE, Hardy-Weinberg Equilibrium.

**Table 2:** Pooled Results for Association of *MTHFR 677C>T* Polymorphism with Risk of Ovarian and Cervical Cancer

Subgroup	Genetic Model	Type of Model	Heterogeneity		Odds Ratio				Publication Bias	
			I^2^ (%)	P_H_	OR	95% CI	Z_test_	P_OR_	P_Beggs_	P_Eggers_
Ovarian Cancer										
Overall	T vs. C	Random	99.38	≤0.001	0.894	0.421-1.896	-0.293	0.770	0.212	0.388
	TT vs. CC	Random	68.02	0.001	1.178	0.894-1.551	1.165	0.244	0.275	0.290
	TC vs. CC	Fixed	26.73	0.190	1.059	0.978-1.147	1.402	0.161	1.000	0.798
	TT+TC vs. CC	Random	50.52	0.027	1.087	0.956-1.236	1.276	0.202	0.640	0.414
	TT vs. TC+CC	Random	61.22	0.004	1.110	0.876-1.406	0.862	0.389	0.350	0.295
Ethnicity										
Caucasian	T vs. C	Random	99.59	≤0.001	0.634	0.240-1.676	-0.919	0.358	0.071	0.737
	TT vs. CC	Fixed	10.34	0.350	0.975	0.854-1.113	-0.377	0.706	0.763	0.106
	TC vs. CC	Fixed	15.31	0.313	1.032	0.948-1.123	0.728	0.467	0.548	0.543
	TT+TC vs. CC	Fixed	21.59	0.265	1.018	0.939-1.103	0.429	0.668	0.367	0.339
	TT vs. TC+CC	Fixed	0.00	0.423	0.958	0.845-1.085	-0.675	0.500	1.000	0.127
Asian	T vs. C	Fixed	9.75	0.344	1.493	1.259-1.772	4.595	≤0.001	0.734	0.351
	TT vs. CC	Fixed	0.00	0.578	2.818	1.857-4.277	4.869	≤0.001	0.308	0.377
	TC vs. CC	Fixed	11.47	0.335	1.300	1.023-1.651	2.147	0.032	0.734	0.880
	TT+TC vs. CC	Fixed	3.27	0.376	1.483	1.183-1.859	3.415	0.001	1.000	0.547
	TT vs. TC+CC	Fixed	0.00	0.608	2.329	1.575-3.445	4.233	≤0.001	1.000	0.324
Cervical Cancer										
Overall	T vs. C	Random	73.78	≤0.001	1.132	0.956-1.341	1.434	0.151	0.620	0.232
	TT vs. CC	Random	49.57	0.013	1.212	0.924-1.590	1.388	0.165	0.964	0.802
	TC vs. CC	Random	77.11	≤0.001	0.985	0.755-1.284	-0.113	0.910	0.843	0.438
	TT+TC vs. CC	Random	79.60	≤0.001	1.095	0.842-1.423	0.677	0.498	0.752	0.215
	TT vs. TC+CC	Random	83.79	≤0.001	1.410	0.913-2.176	1.551	0.121	0.620	0.867
Ethnicity										
Caucasian	T vs. C	Fixed	45.38	0.160	1.071	0.891-1.287	0.733	0.464	1.000	0.431
	TT vs. CC	Fixed	8.40	0.336	0.996	0.637-1.559	-0.016	0.987	1.000	0.439
	TC vs. CC	Fixed	27.78	0.250	1.197	0.930-1.540	1.393	0.164	1.000	0.413
	TT+TC vs. CC	Fixed	44.89	0.163	1.162	0.912-1.480	1.214	0.225	1.000	0.406
	TT vs. TC+CC	Fixed	0.00	0.573	0.913	0.594-1.403	-0.415	0.678	1.000	0.470
Asian	T vs. C	Random	77.58	≤0.001	1.173	0.958-1.438	1.542	0.123	0.427	0.119
	TT vs. CC	Random	55.31	0.008	1.295	0.944-1.776	1.602	0.109	0.854	0.530
	TC vs. CC	Random	79.26	≤0.001	0.967	0.705-1.325	-0.211	0.833	0.945	0.264
	TT+TC vs. CC	Random	82.20	≤0.001	1.119	0.816-1.532	0.697	0.486	0.427	0.116
	TT vs. TC+CC	Random	86.09	≤0.001	1.594	0.961-2.642	1.806	0.071	0.582	0.924

*Statistical Analysis*

The strength of association between *MTHFR 677C>T* polymorphism and ovarian and cervical cancers was assessed by using odds ratios (ORs) and 95% confidence intervals (CIs). The P-value of the pooled ORs was considered significant if less than 0.05, which was examined by Z-test. The pooled ORs were calculated under all five genetic models, i.e., allele (T vs. C), homozygote (TT vs. CC), heterozygote (TC vs. CC), dominant (TT+TC vs. CC), and recessive (TT vs. TC+CC), in which the ‘’C’’ represents the major allele and the ‘’T’’ represents the minor allele. The Cochran’s Q-test was used to access the between-study heterogeneity. Moreover, the effects of heterogeneity was we quantified using I2 statistic (ranges from 0 to 100%), in which detected variations among studies due to heterogeneity rather than chance (I2= 0-25%, no heterogeneity; I2=25-50%, moderate heterogeneity; I2=50–75%, large heterogeneity; I2=75-100%, extreme heterogeneity) (Higgins 2003, Hippel 2015). A chi-square test was used to determine Hardy-Weinberg equilibrium (HWE) in controls, which p-value less than 0.05 was representative of statistical significance. Subgroup analyses were performed to explore possible sources of heterogeneity by ethnicity, source of controls, genotyping method and HWE status. Sensitivity analyses were performed by sequential removal of each study and by excluding those studies deviation from HWE to test the stability and reliability of the results. Visual inspection of asymmetry in funnel plots and Begg’s rank correlation statistically were used to test whether publication bias existed or not, in which P<0.05 was considered to be represented of statistically significant. All statistical analyses were performed using the Comprehensive Meta-Analysis (CMA) software version 2.0 (Biostat, USA). Two-sided P<0.05 was considered statistically significant.

## Results

*Characteristics of Studies*

The flow diagram of study selection process was presented in Figure 1. According to the initial searches, 123 studies were identified, which after removing duplicates and irrelevant studies, there were 69 studies left. Then, the titles and abstracts of the remaining articles were reviewed, 51 full-text articles were considered eligible. After carefully reviewing the remaining studies, 24 of them were excluded because did not reported sufficient data, were not case-control studies, overlapped by other studies, and not relevant to the *MTHFR 677C>T* polymorphism (Figure 1). Finally, 27 case-control studies with 7856 ovarian and cervical cancer cases and 11,263 controls were included. Among these studies, eleven case-control studies with 4990 cases 7730 controls were on ovarian cancer (Jakubowska et al., 2007; Wu et al., 2007; Terry et al., 2010; Prasad and Wilkhoo, 2011; Webb et al., 2011; Gao et al., 2012; Pawlik et al., 2012; Zhang et al., 2012; Özkılıç et al., 2016) and 16 case-control studies with 2,866 cases 3,533 controls were on cervical cancer (Lambropoulos et al., 2003; Sull et al., 2004; Zoodsma et al., 2005; Kang et al., 2005; Delgado-Enciso et al., 2006; Ma et al., 2006; Shekari et al., 2008; Nandan et al., 2008; Kohaar et al., 2010; Tong et al., 2011; von Keyserling et al., 2011; Yang et al., 2011; Mostowska et al., 2011; Prasad and Wilkhoo, 2011; Hajiesmaeil et al., 2016; Gong et al., 2018). The main characteristics of included studies were listed in Table 1. The studies were published from 2004 to 2018, and the sample sizes in cases groups ranged from 22 to 1,638. For ovarian cancer, seven studies were conducted in Caucasians and four in Asians. For cervical cancer, eleven studies were conducted in Asians, four in Caucasian, and one study in mixed population. All the 27 included studies were case-control studies, 24 of them were in a Hospital-Based (HB) and the remaining was Population-Based (PB) design. Five different genotyping methods were used including: PCR-PFLP, TaqMan, MassARRAY, SNapShot and LDR-PCR. The genotype distribution of the healthy subjects in all included studies was in agreement with the Hardy-Weinberg equilibrium (HWE), except one study for cervical cancer (Table 1).

*Quantitative Synthesis*

*Ovarian Cancer*

The results of meta-analysis for association between the *MTHFR 677C>T* polymorphism and ovarian cancer were listed in Table 2. In overall, pooled data showed that the *MTHFR 677C>T* polymorphism did no significantly associated with an increased risk of ovarian cancer under all five genetic models, i.e., allele (T vs. C: OR = 0.894, 95% CI 0.421-1.896, p = 0.770), homozygote (TT vs. CC: OR = 1.178, 95% CI 0.894-1.551, p = 0.244); heterozygote (TC vs. CC: OR = 1.059, 95% CI 0.978-1.147, p = 0.161, Figure 2A), dominant (TT+TC vs. CC: OR = 1.087, 95% CI 0.956-1.236, p = 0.202), and recessive (TT vs. TC+CC: OR = 1.110, 95% CI 0.876-1.406, p = 0.389). When stratified by ethnicity, there was a significant association between the *MTHFR 677C>T* polymorphism and an increased risk of ovarian cancer in Asians under all five genetic models, i.e., allele (T vs. C: OR = 1.132, 95% CI 1.259-1.772, p ≤0.001), homozygote (TT vs. CC: OR = 1.212, 95% CI 1.857-4.277, p ≤0.001); heterozygote (TC vs. CC: OR = 0.985, 95% CI 1.023-1.651, p = 0.032), dominant (TT+TC vs. CC: OR = 1.095, 95% CI 1.183-1.859, p = 0.001), and recessive (TT vs. TC+CC: OR = 1.410, 95% CI 1.575-3.445, p ≤0.001), but not in Caucasians (Table 2).

*Cervical Cancer*

Table 2 also summarizes the results of association between the *MTHFR 677C>T* polymorphism and cervical cancer. Pooled data failed to show a significant association between *MTHFR 677C>T* polymorphism and risk of cervical cancer under all five genetic models, i.e., allele (T vs. C: OR = 1.132, 95% CI 0.956-1.341, p = 0.151), homozygote (TT vs. CC: OR = 1.212, 95% CI 0.924-1.590, p = 0.165); heterozygote (TC vs. CC: OR = 0.985, 95% CI 0.755-1.284, p = 0.910), dominant (TT+TC vs. CC: OR = 1.095, 95% CI 0.842-1.423, p = 0.498, Figure 2B), and recessive (TT vs. TC+CC: OR = 1.410, 95% CI 0.913-2.176, p = 0.121). Similarly, stratified analysis by ethnicity did not show a significant association between the *MTHFR 677C>T* polymorphism and cervical cancer under all five genetic models in Asians and Caucasians (Table 2).

*Between-Study Heterogeneity Test*

In the current meta-analysis, there was obvious between-study heterogeneity under four genetic models for ovarian cancer and all five genetic models for cervical cancer in overall population (Table 2). Therefore, we performed subgroup analyses by ethnicity to assess the potential source of between-study heterogeneity. In subgroup analysis, between-study heterogeneity was disappeared for ovarian cancer in the Caucasian and Asian population, as well as in the Caucasian subgroup for cervical cancer. The subgroup analysis results showed that ethnicity might be the major source of between-study heterogeneity for both ovarian and cervical cancer in the current meta-analysis.

*Sensitivity Analysis*

Moreover, sensitivity analysis was performed to assess the influence of each independent study on the pooled ORs by the sequential removal of each individual study form the analysis. However, the results of the sensitivity analysis for both ovarian cancer and cervical cancer did not materially changed by removing any of each individual study. Moreover, sensitivity analysis was carried out by excluding the HWE-violating study (Nandan et al., 2008) for cervical cancer. However, excluding the study did not significantly affect the pooled ORs for cervical cancer, indicating the robustness and reliability of this meta-analysis.

*Publication Bias*

Begg’s and Egger’s tests were used to examine the potential publication bias in assessment of the association of *MTHFR 677C>T* polymorphism with ovarian and cervical cancer risk in all genetic models. No asymmetry was observed in the Begg’s rank correlation among the studies on ovarian and cervical cancer. Figure 3 showed the shape of the Begg’s funnel plots for association between *MTHFR 677C>T* polymorphism and risk of cervical cancer in recessive model (TT vs. TC+CC). Moreover, the Egger’s linear regression test did not show any statistical evidence of publication bias among the studies on ovarian and cervical cancer (Table 2).

## Discussion

MTHFR is an important enzyme which has an important role in the regulation of methionine and homocysteine levels in folate metabolism. The *MTHFR 677C>T* polymorphism is one of the most studied functional polymorphism in cancer development, which could reduce the production of *MTHFR* and affect enzyme activity (Kamali et al., 2018). Thus, the current meta-analysis was performed to evaluate the association of *MTHFR 677C>T* polymorphism with susceptibility to ovarian and cervical cancers in women. Finally, 27 case-control studies including eleven studies on ovarian cancer and 16 studies on cervical cancer were selected. Therefore, the current meta-analysis was the largest scale study so far on *MTHFR 677C>T* polymorphism association with ovarian and cervical cancers. This meta-analysis pooled data revealed that the *MTHFR 677C>T* polymorphism was not associated with an increased risk of ovarian and cervical cancers in overall population. The negative results in this pooled analysis agrees with the previous meta-analysis on cervical cancer (Yi et al., 2016). However, He and Shen (2017) in meta-analysis of eight studies found that the *MTHFR 677C>T* polymorphism was associated with ovarian cancer risk. The main strength of this meta-analysis in comparison with the previous meta-analyses was the total number of cases and healthy controls selected. Therefore, this meta-analysis more power to detect the small effects of the polymorphism than previous studies. However, we recommend increasing the sample size in future studies in order to increase the power to detect small effects of the *MTHFR 677C>T* polymorphism on risk of ovarian and cervical cancers.

When stratified analysis by ethnicity was performed the results showed that the *MTHFR 677C>T* polymorphism was significantly associated with ovarian cancer in Asians, but not in Caucasians. Similarly, He and Shen (2017) showed that the *MTHFR 677C>T* polymorphism is a risk factor for ovarian cancer and also breast cancer in Asians. It seems that due to the some genetic and environmental differences between Asian and Caucasian populations, the *MTHFR 677C>T* polymorphism might play a different role in the development of ovarian cancer in the two populations. However, the subgroup analysis did not show a significant association between *MTHFR 677C>T* polymorphism and cervical cancer by ethnicity. However, the previous meta-analysis revealed that *MTHFR 677C>T* polymorphism was significantly associated with cervical cancer in Asians (Yi et al., 2016).

Between-study heterogeneity refers to the variation in study results between different studies, which could affects pooled results of a meta-analysis (Kamali et al., 2017) and a significant problem when interpreting of a meta-analysis (Forat-Yazdi et al., 2017; Jafari-Nedooshan et al., 2017). Several factors such as sample size, ethnicity, source of controls, genotyping methods, participants demographic and lifestyle might lead to the heterogeneity among studies (Mehdinejad et al., 2017; Yazdi et al., 2017). Thus, to explore the potential sources of heterogeneity among studies, we conducted subgroup analyses by ethnicity, cancer, source of control, HWE and genotyping methods. However, the subgroup analysis results showed that only ethnicity was the main source of heterogeneity in this meta-analysis.

To the best knowledge, this meta-analysis was the most comprehensive and convicting on the association of the *MTHFR 677C>T* polymorphism with susceptibility to ovarian and cervical cancer. However, there were some limitations in our meta-analysis which must be described. First, we have only focused those published studies in English and Chinese in the current meta-analysis. Second, in the current meta-analysis the number of studies and the sample size in the studies by other ethnicities such as Africans, Latinos and mixed populations were small. Therefore, the lack of power due to the small number of studies leaves it an open field by ethnicity. Third, our meta-analysis was not adjusted by the potential confounders, such as age, gender and lifestyle, because not all of the studies reported adjusted ORs potential confounders. Finally, the potential effect of *MTHFR 677C>T* polymorphism might be affected by gene-gene and gene-environment interactions. However, due to the lack of original data limited further evaluation of potential gene-gene and gene-environment interactions and also interactions of other polymorphisms of* MTHFR* gene.

In summary, this meta-analysis demonstrated that the *MTHFR 677C>T* polymorphism was not associated with an increased risk to ovarian and cervical cancer in overall population. However, *MTHFR 677C>T* polymorphism was significantly associated with ovarian cancer in Asians, but not in Caucasians. Moreover, considering the limitations of the study, large well-designed studies from different ethnicities should be conducted to provide a better understanding of the association of *MTHFR 677C>T* polymorphism with risk of ovarian and cervical cancer.
